# Environmental Benefits of Electronic Table of Contents of Journals Over Print

**DOI:** 10.7759/cureus.47907

**Published:** 2023-10-29

**Authors:** Oliver Ralph, Gwyneth Sullivan, Edie Chan, Oyedolamu K Olaitan

**Affiliations:** 1 General Surgery, Rush University Medical Center, Chicago, USA; 2 Transplantation, Rush University Medical Center, Chicago, USA; 3 Surgery, Rush University Medical Center, Chicago, USA

**Keywords:** online content, online media, publication trends, environment friendly, environment, hospital environment

## Abstract

Recent discussion has driven debate on the best format for journals to deliver content to their readers. Traditional dogma necessitated a physical print copy, which was sent to subscribers automatically and came with the benefits of ease of use and familiarity. With the passage of time, electronic tables of contents, with or without the option for a print copy, have been used in lieu to save cost and environmental concerns and to allow content to be consumed in a more convenient, tidier way.

## Editorial

With the debate of electronic versus print publication ongoing, the rising cost of postage and labor has provided momentum for a digital switch, particularly with smaller publications. From a consumer’s perspective, we believe that the balance of considerations favors the electronic format, with an electronic table of contents (eTOC). Of course, there is significant waste and cost to large print editions. However, traditional reticence to a switch has focused on a concern that subscribers may be less inclined to consume content in electronic form, with emails ignored and a lack of a physical reminder on one’s desk. Indeed, there are known downsides to a switch to eTOC. For such content to be consumed, readers should be willing to search through inboxes for it. Spam email folders are a concern as is the requirement to maintain up-to-date email databases for readers, while annotation is clearly more difficult with electronic information. Such concerns are well founded, but we believe they are ultimately misplaced. The electronic publication provides the flexibility to consume material in multiple locations at different times without the need to transport a physical copy while also giving the convenience of searching for relevant topics or citations. If a reader is likely to consume content in printed form, it stands to reason that he/she will know where and how to find the eTOC. Experience shows that it detaches the link between consumption and location and is thus immensely valuable, particularly to practicing members, leading to an increase in content consumption as compared to a hard copy. In addition, at our institution, print editions are typically piled high, partially read, or at times, not read at all, resulting in significant waste. It is the included delivery of printed copies with a membership subscription that perpetuates this problem and is something that could be remedied with electronic copies with an opt-in option for print. This would take advantage of the many cost and convenience benefits of eTOC while preserving the preference of those who enjoy the physical touch of the written word.

Two primary concerns may relate to the effect of the transition to electronic notification on membership subscriptions and the journal’s impact factor. With the paucity of literature on the topic, all that can be done is to examine the success of journals that have gone this way. One such example is the *Journal of the American College of Surgeons* (*JACS*), which, like the *American Journal of Transplantation* through the American Society of Transplant Surgeons and the American Society of Transplantation, employs an active membership base through the American College of Surgeons and strong online resources. *JACS* continues to enjoy high relevance while not providing copies in print without a separate subscription. Indeed, *JACS* emails a monthly table of contents (TOC) to its members with links to highlighted publications found within. This allows for rapid digestion of publications of interest and therefore comes with high readership within our department - it seems the speed and efficiency of an eTOC is directly proportional to the time spent reading the related content [1]. In addition, derivations of *Nature*, *Oncogenesis*, and *Critical Care* journals, to name but a few, have all made the change without negative consequences, while clinically based journals, particularly in dermatology, psychology, and pathology, were also among the early trendsetters and continue to enjoy academic success [2,3]. Perhaps, usability for members coupled with a growing demand for environmentalism that may necessitate the change is more important than subscription rates or indeed the controversial impact factor.

The recent commitment of Rush University Medical Center (RUMC) to reduce greenhouse gas emissions by 50% and have net zero emissions by 2050 is part of the motivation for this communication. The Department of Health and Human Services Health Care Sector Climate Pledge currently has more than 60 institutions signed up to it [4]. This represents a broad and pervasive attitude of environmentalism that emanates from every department at RUMC. In the operating room (OR), we have a greening OR team that has been working toward reducing environmental impact through three main strategies: anesthetic practices, waste reduction, and energy efficiency [5]. Desflurane use is limited, single-use devices reduced, operative trays simplified, and entire teams created that are dedicated to waste reduction and increased efficiency. With such a recent focus on waste reduction, supply chain efficiency, and green education, it naturally follows that our transplant faculty are fully behind reducing their use of printed journals and favoring a move to online resources. Indeed, 32 million trees in the USA are used annually in literature production of all types. Each 20-page printed journal article expends 1.2 kg of CO_2_ and requires the use of 6.4 L of fresh water [6]. When extrapolated nationwide and taking into account the high percentage of printed issues that are never consumed, the scale of such unnecessary waste becomes clear.

The purpose of this communication is to challenge readers to consider the future of how literature is consumed from both an environmental and practical standpoint. It is our opinion that an eTOC provides not just more convenience but significant benefits in terms of waste and cost while preserving the relevance and scope of the respective journals. It should be noted that this communication is based on anecdotal evidence and the experiences of faculty at only one institution. However, the themes remain valid across institutions. Based on the listed environmental work RUMC is doing, it remains difficult to reconcile this waste in the face of journals that provide readily digestible content in an electronic format that can be consumed at any local at a moment's notice.
